# The Impact of Shear and Elongational Forces on Structural Formation of Polyacrylonitrile/Carbon Nanotubes Composite Fibers during Wet Spinning Process

**DOI:** 10.3390/ma12172797

**Published:** 2019-08-30

**Authors:** Hamideh Mirbaha, Parviz Nourpanah, Paolo Scardi, Mirco D’incau, Gabriele Greco, Luca Valentini, Silvia Bittolo Bon, Shahram Arbab, Nicola Pugno

**Affiliations:** 1 Department of Textile Engineering, Amirkabir University of Technology, 15875-4413 Tehran, Iran; 2 Laboratory of Bio-inspired & Graphene Nanomechanics, Department of Civil, Environmental and Mechanical Engineering, University of Trento, 38123 Trento, Italy; 3 Department of Civil, Environmental and Mechanical Engineering, University of Trento, 38123 Trento, Italy; 4 Civil and Environmental Engineering Department, University of Perugia and INSTM Research Unit, 05100 Terni, Italy; 5 Advanced Textile Materials and Technology Research Institute, Department of Textile Engineering, Amirkabir University of Technology, 15875-4413 Tehran, Iran; 6 School of Engineering and Materials Science, Queen Mary University of London, London E1 4NS, UK; 7 Ket-Lab, Edoardo Amaldi Foundation, 00133 Rome, Italy

**Keywords:** PAN/CNT composite fibers, multi walled CNT, wet spinning, shear, elongation, mechanical properties, crystalline structure

## Abstract

Wet spinning of polyacrylonitrile/carbon nanotubes (PAN/CNT) composite fibers was studied and the effect of spinning conditions on structure and properties of as-spun fibers influenced by the presence of CNTs investigated. Unlike PAN fibers, shear force had a larger effect on crystalline structure and physical and mechanical properties of PAN/CNT composite fibers compared to the elongational force inside a coagulation bath. Under shear force CNTs induced nucleation of new crystals, whereas under elongational force nucleation of new crystals were hindered but the already formed crystals grew bigger. To our knowledge, this key effect has not been reported elsewhere. At different shear rates, strength, Young’s modulus and strain at break of PAN/CNT as-spun fibers were improved up to 20% compared to PAN fibers. Application of jet stretch had less influence on physical and mechanical properties of PAN/CNT fibers compared to PAN fibers. However, the improvement of interphase between polymer chains and CNTs as a result of chain orientation may have contributed to enhancement of Young’s modulus of jet stretched composite fibers.

## 1. Introduction

Carbon nanotubes (CNTs) have numerous applications due to their extraordinary properties, among which production of composite fibers based on polymer/CNT with ability to improve physical, mechanical, thermal, electrical and sound absorption properties can be noted [1,2,3,4,5,6,7,8,9]. Production of polyacrylonitrile (PAN)/CNT composite fibers with potential to be used as carbon fiber precursor and to improve mechanical properties of carbon fibers has attracted attention in recent years [6,10,11,12,13]. Since the parameters affecting structure formation during spinning process are diverse, successful production of PAN/CNT composite fibers with desired properties requires intensive research and correlation of the results. If production conditions are not controlled, CNTs may exist as aggregations, prohibiting orientation of polymers. Therefore, lower structural order, more defects and lack of crystalline interphase can be expected. In such conditions, exploiting desirable properties of CNTs inside fibers is not possible. There has been some research in recent years on PAN/CNT composite fibers, with a focus on the effect of different CNT types, content and post-processing on the structure and properties of final PAN/CNT fibers or their conversion to carbon fibers [1,6,10]. However, the structure formation of as-spun fibers in early stages of production in a coagulation bath has not been studied intensively and in detail. Understanding the effect of spinning conditions on the structure of as-spun fibers can give a bright image to design the wet spinning process more effectively. Thus, studying the structure formation of PAN/CNT composite fibers in early stages of production and the effect of different parameters in the spinning process is the focus of this paper.

The most important step in determination of fiber properties is the structure formation during the spinning process [14,15,16]. In the wet spinning process, polymer solution is extruded through a spinneret into a coagulation bath. The formed filaments are jet stretched by take-up rollers and then are subjected to washing, post-drawing and drying processes [14,15,17]. When the spinning dope is passing through the spinneret, shear force is applied on polymer chains. Polymer chains are straightened under shear, leading to a decrease in internal friction of the spinning fluid [4,18]. The studies on melt spinning of polypropylene in the presence of CNTs show that the shear stress is also transferred to nanotubes, orientating them in the direction of shear flow [19]. When applied stress on CNTs exceeds a critical agglomeration strength, it can break the aggregations and separated nanotubes can orient more easily than their aggregations [19].

Improvement in orientation of polymer chains can be done by applying stretch on fibers in different production stages including jet stretch and drawing of as-spun fibers [20,21,22]. During the stretching process of solidifying spinning dope inside the coagulation bath—jet stretch stage-the rheological resistance is small making the orientation of macromolecules easier [4]. In the wet spinning process, jet stretch and shear stress coexist simultaneously and influence the structure formation and polymer chain orientation. Therefore, the necessity of detailed studies on the effect of these forces on the formation, development, and alignment of the structure and properties of the PAN/CNT as-spun fibers is evident. It is widely accepted that alignment of CNTs along fiber direction is important in order to take advantages of potential improvement in structure and properties of composite fibers [2]. Mai et al. [23] studied the effect of extension from take up rollers on the structure and properties of melt spun polyamide 66-multi walled CNTs (MWCNTs) composite fibers. They observe that high draw ratio not only can improve the dispersion of MWCNTs inside the polymer matrix, but also it can increase the orientation of fibers as well as interfacial adhesion between the matrix and MWCNTs, leading to improvement in mechanical properties of fibers. Mikolajczyk et al. [4] suggested that the presence of CNTs among polymers, similar to the softener effect, can facilitate the slippage of polymer chains and reduce the internal friction of the fluid. Many researchers have studied the effect of shear and elongation flows and their simultaneous impact on the structure formation and chain orientation of PAN fibers. However, the presence of CNTs among PAN can lead to different chain behavior under shear and elongational forces, which is not studied intensively and comprehensively. 

Studying the effects of shear and elongational forces at different stages of fiber production, from spinning dope preparation to wet spinning and jet stretching on the structure evolution of as-spun PAN/CNT fibers can be a part of a road-map for controlling spinning parameters in order to improve the PAN/CNT fiber structure and properties. It can help to proceed on the route of improving the mechanical properties of PAN/CNT based carbon fibers. Accordingly, morphology, physical and mechanical properties and crystalline structure of as-spun PAN/CNT composite fibers at different stages of the wet spinning process have been studied in this paper. The effect of shear rates and jet stretch ratios on the fiber structure as well as the effect of the presence of CNTs on structure formation and evolution compared to PAN fibers have been investigated.

## 2. Methods

### 2.1. Materials, Ingredients, Synthesis

PAN powder with viscosity molecular weight of 112,000 g/mol containing comonomers of methyl acrylate and sodium methallyl sulfonate was obtained from Polyacryl Corp., Iran. Analysis grade dimethyl formamide (DMF) solvent, (Merck Co., Darmstadt, Germany) was used as received. Multi walled carbon nanotubes (MWCNTs) (Research Institute of Petroleum Industry, Tehran, Iran) with the average length of 10 micron, average diameter of 10–30 nanometers and purity over 95% were used.

### 2.2. Spinning Dope Preparation

For production of reference PAN fibers, PAN/DMF spinning dope was prepared from 20% w/v solution of PAN in DMF solvent. For production of PAN/CNT composite fibers, first 20% w/v solution of PAN in DMF was prepared. Then a suspension of CNT in 400 mL of DMF solvent was prepared with a concentration of 0.75 wt% of CNT in proportion to the polymer weight. The concentration of 0.75% for CNT was chosen based on the literature and the optimization experiments regarding the dispersion of CNTs in a polymer solution. For the purpose of dispersion of CNT in DMF, a UP200S ultrasonic homogenizer (Hielscher Ultrasonics GmbH, Teltow, Germany) was utilized for 30 minutes. Then the polymer solution was gradually introduced to the dispersion under shear force applied from a mechanical stirrer until a homogenous solution was obtained. The ultrasonication process was again continued for 15 minutes. According to Zhao et al. [24], polymers can permeate into bundles of CNTs through ultrasonication and prevent the van der Waals adherence between one CNT and other CNT bundles. As a result, a thicker bundle of CNTs is divided into thinner sub-bundles and even up to single nanotubes. Zhao et al. [24] also observed higher stability of CNT dispersion when the polymer exists in the solution during ultrasonication. After ultrasonication, the extra solvent was evaporated under shear force and heat until the PAN/CNT/DMF solution with a concentration of 20% PAN and 0.75% CNT was obtained. 

### 2.3. Wet Spinning

**Figure 1:**
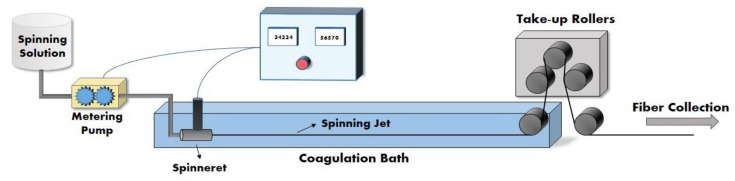
Schematic diagram of the wet spinning machine used in the present work.

Composite PAN/CNT and reference PAN fibers were wet spun using a laboratory spinning machine. Figure 1 shows a schematic diagram of the wet spinning machine used in the present work. The spinning dope was pumped at different speeds to a one-hole spinneret with a diameter of 200 microns using a metering pump and then was extruded into a coagulation bath containing water as nonsolvent at a temperature of 20 °C. The freely spun fibers were obtained by taking fibers directly out of the coagulation bath and then washing and drying at room temperature. The coagulated fibers at different jet stretch ratios, (2, 2.5, 3 and 3.5) were collected at the take-up rollers by changing the velocity of the take-up rollers for the same extrusion velocity of the spinning solution. Jet stretch ratios are defined as take-up velocity of the coagulated fiber to the extrusion velocity of the spinning solution. The collected fiber samples were soaked in and washed thoroughly with water and finally, they were allowed to dry naturally in air.

In Table 1, denotation and different conditions for the production of reference PAN and composite PAN/CNT fibers are listed. In the fiber denotation, P and PC stand for PAN and PAN/CNT, respectively. The first numbers are equivalent shear rates inside the spinneret (s^−1^ ×10^−3^) as listed in Table 1. The second numbers of 2, 2.5, 3 and 3.5 coming after the letter *D* are the jet stretch ratios applied on fibers inside the coagulation bath. The fiber codes without a second number are freely spun fibers. The equivalent shear rate inside the spinneret ($\gamma^{{}^{\circ}}$) in unit of inverse seconds (s^−1^) is calculated as [18]:

(1) $$\gamma^{{}^{\circ}}=32Q/\left(\pi D^{3}\right)$$

where *Q* is volumetric flow rate and *D* is the diameter of the spinneret hole. This equation simply assumes that the polymer dope is Newtonian and it is in fully developed flow.

### 2.4. Fiber Characterization

The fiber diameter was measured at numerous points along fiber length using a Microphot FXA optical microscope (Nikon, Tokyo, Japan) at magnification of 50×. To estimate the die swell ratio, spinning dope was extruded to a coagulation bath freely without applying jet stretch and the diameter of fiber cross section was measured by Microphot FXA microscope (Nikon, Tokyo, Japan). The die swell ratio was obtained from dividing this diameter by the diameter of the spinneret hole [16,25]. The linear density was calculated by measuring the mass of different lengths of fibers with a GR-200 balance (A and D Balance, Tokyo, Japan). The average values of linear density were reported in the unit of tex (weight in grams of 1000 meters of fibers). The overall porosity of fibers was estimated by measuring the mass (via weight) and calculating the volume of fibers at numerous one-meter long replicates of fibers [26]:

(2) $$Overall\;porosity\;\left(\%\right)=(\left(\pi R^{2}L-m\rho^{-1}\right)/\pi R^{2}L)\times 100$$

where *R*, *L*, *m* and *ρ* are radius, length, mass of fibers and density of polymer, respectively.

The glass transition temperature (*T_g_*) of selected fibers was measured using a DSC Q200 (TA Instruments, FC, USA) with dynamic scans between 60 °C and 160 °C at a heating rate of 2 °C/min.

The microscopic images of Pt coated cross sections of fibers at magnifications of 500× and 7000× were obtained using a SUPRA™ 40 VP Field Emission Scanning Electron Microscope (FESEM) (Zeiss, Oberkochen, Germany) at 2 kV. The images at magnification of 150,000× were captured using a MIRA3 FESEM (Tescan, Brno, Czech Republic) at 15 kV. The intact cross sections of fibers were obtained by cutting the samples in liquid nitrogen. 

The crystalline structure of PAN and PAN/CNT fibers was studied using an X-ray diffractometer (Panalytical X’Pert MRD, Malvern, United Kingdom) operated at 40 kV and 40 mA using Co K-α radiation (wavelength: 1.790 Angstrom). Bundles of fibers were placed on the sample holder. The scanning range of 10°–48° (2θ) with a scanning step of 0.15° and counting time of 25 s/step was used. To extract data from XRD patterns, the peak resolving method in Origin 2017 software (OriginLab, Massachusetts, USA) based on three Lorenz functions was utilized to get the best fit for the experimental diffraction curve. The intense peak at 2θ∼17° was used to calculate the crystalline parameters. The plane spacing (*d*) was calculated from Bragg’s formula and lateral crystal size (*L_c_*) was obtained using Scherrer equation [25,27]:

(3) $$L_{C}=K\lambda /(\beta cos\theta )$$

where *K* is a constant with value of 0.89 and *β* is the full width at half maximum intensity (FWHM).

Among different methods for the calculation of relative degree of crystallinity, the Gupta–Singhal method was more reasonable and repeatable for the samples of this research. Therefore, this method was used here [28]:

(4) $$Crystallinity\;\left(\%\right)=\;A_{crystal}/A_{total}\times 100$$

where *A_crystal_* is the area of crystalline peaks and *A_total_* is the total area of diffraction over the entire measurement range after the background subtraction.

Mechanical properties of PAN and PAN/CNT single filaments were measured using a Universal testing machine MIDI 10 (Messphysik Materials testing GmbH, Fürstenfeld, Austria) using a 10 N load cell. The distance between two grips was 1 cm and the elongation rate was 0.1 mm/s (based on standard D3822). Each mechanical property was reported as the average value of at least 10 test results, and the standard deviation and coefficient of variation (CV%) were calculated. It needs to be mentioned that diversity of data between different repeats of a sample was low. Breaking tenacity was calculated from dividing the load at break by linear density of fibers. Tex was used as linear density that is defined as the mass in grams of 1000 meters of fibers. The Young’s modulus (cN/tex) was obtained from the initial slope of the specific stress–strain curve. The toughness was calculated from the area under the tenacity–strain curve up to the failure point.

The electrical properties were measured using Keithley 4200-SCS having two probes with 25 mm distance (Keithley semiconductor characterization system, OH, Unites States). The electrical conductivity σ (S/cm) was calculated as [29]:

(5) σ=IL/(VA)

where *I*, *L*, *V* and *A* are electrical current, sample length, voltage and fiber cross section area, respectively.

## 3. Results and Discussion

### 3.1. Jet Swell Ratio

**Figure 2:**
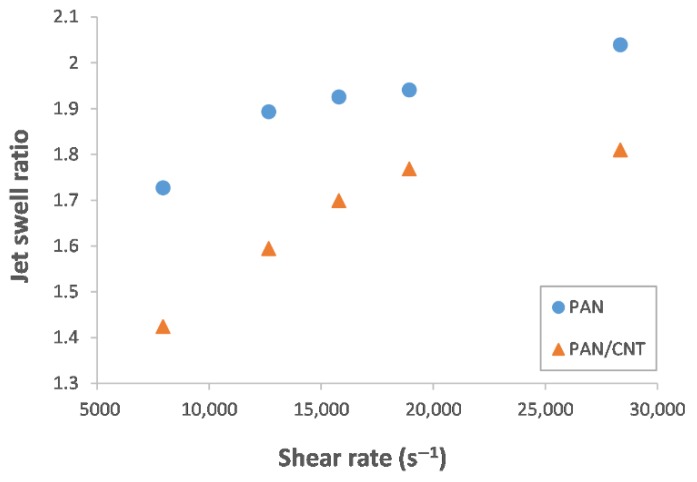
Jet swell ratio of PAN and PAN/CNT fibers at different shear rates.

When spinning dope is extruded freely from a spinneret into a coagulation bath, radial expansion of the thread takes place, resulting in a loss of orientation of polymer chains. This phenomenon that is called jet swell is a result of viscoelasticity of polymer solutions. It happens because of the energy storage from shear forces applied on spinning dope during its entrance and exit from the spinneret hole [16,19,25,30]. In Figure 2, the die swell ratio as a function of shear rate for PAN and PAN/CNT fibers is shown. With an increase in shear rate the jet swell ratio increases. 

The jet swell ratio of PAN/CNT fibers is less than PAN fibers. As a result, polymer chains in composite fibers can preserve their orientation along the fiber direction better. Choi et al. [30] also observed lower jet swell with addition of CNT to PAN fibers. It seems that presence of CNTs inside PAN solution, decreases the elastic recovery of PAN chains, affects the chain mobility and reduces the internal friction [30]. In addition, the decrease in jet swell ratio of a PAN solution in presence of CNTs may indicate the formation of an interphase between polymer and nanotubes. 

When spinning dope is extruded to a coagulation bath under certain tension applied by jet stretch, the jet swell can be limited or eliminated [16,25]. Having a clear understanding of the jet swell effect, it is necessary to optimize jet stretch ratio to obtain proper fiber structure. Considering the estimated values for jet swell ratio at shear rate of 8000 s^−1^ (Figure 2), a jet stretch ratio of about two is required to overcome the jet swell of PAN fibers and to obtain a fiber with diameter of 200 microns (the same as the spinneret hole diameter). For PAN/CNT composite fibers, this ratio is about 1.4, due to the role of CNTs in reducing the jet swell effect. Thus, CNTs help to overcome jet swell at lower jet stretch ratios. Therefore, at jet stretch ratio of two and higher used in this research, the jet swell effect in both PAN and PAN/CNT fibers was eliminated and the diameter of fibers was less than the spinneret hole diameter. In addition, due to lower jet swell ratio in PAN/CNT composite fibers, the stretching force applied on polymer chains at the same jet stretch ratio was higher than PAN fibers. This could possibly lead to higher chain orientation in PAN/CNT fibers.

### 3.2. Physical Properties

The average diameter, linear density and overall porosity of PAN and PAN/CNT fibers at different shear rates and jet stretch ratios are reported in Table 2. 

#### 3.2.1. Shear Rate Effect

With an increase in the shear rate, the diameter and linear density of PAN and PAN/CNT fibers increased (Table 2). The presence of CNTs among PAN chains slightly decreased the fiber diameter. This was attributed to smaller jet swell ratio in the presence of CNTs (Figure 2) [30]. The diameter of PAN/CNT fibers at different shear rates was about 9%–19% lower than corresponding PAN fibers, whereas the linear density is about 33%–48% less than PAN fibers. Since linear density is the mass per unit length of fibers, this significant discrepancy in the diameter and linear density between PAN and PAN/CNT fibers showed that a structure with higher porosity is formed in PAN/CNT fibers. The pores can be the origin for fiber fracture, with an important effect on mechanical strength of fibers [31]. The calculated overall porosity of fibers at different shear rates in Table 2 also confirmed that the presence of CNTs among polymer chains led to higher porosity of structure. Apparently, CNTs and their aggregations inside spinning dope can act as a hindrance for compactness of structure. Addition of CNTs to PAN fibers led to an increase in *T_g_*. Mahfuz et al. [32] also observed about 5 °C increase in *T_g_* due to addition of CNTs to nylon filaments. This increase in *T_g_* might be due to restrictions in mobility of PAN chains, possibly as a result of formation of a strong interface between PAN and CNTs or crystallization of structure in the vicinity of CNTs [33,34]. 

#### 3.2.2. Jet Stretch Effect

When jet stretch was applied on fibers inside the coagulation bath, the diameter and linear density of PAN and PAN/CNT as-spun fibers decreased considerably (Table 2). Diameter and linear density of PAN/CNT fibers were less than PAN fibers at different jet stretch ratios. The tolerance of PAN/CNT composite fibers against jet stretch was less than PAN, as they could not tolerate a high jet stretch ratio of 3.5. It may be a result of higher stretch forces exerted on polymers due to lower jet swell ratios. In addition, slippage of polymer chains over each other due to lower internal friction in composite fibers may have led to filament breakage during stretching. In addition, presence of nano-additives may have reduced the filament ability to distort in the drawing stage [35]. 

For both PAN and PAN/CNT fibers, increasing the jet stretch ratio resulted in an increase in overall porosity. For reference PAN fibers, applying jet stretch up to a ratio of three decreased the overall porosity compared to corresponding freely spun fibers. This could be due to alignment of polymer chains and fibrils in a more oriented and compact form, thus with fewer pores and voids, as well as radial shrinkage and diameter reduction. Inside a coagulation bath the movement of structural sections of fibers is easier compared to coagulated fibers since fibers are not yet completely solidified. Thus a more compact structure can be formed by stretching. In addition, applying jet stretch reduces the time that fibers remain in a coagulation bath. As a result, the available time for pore growth decreases. The presence of CNTs, however, had different influence on this behavior. Since jet stretch ratios were apparently higher than optimum values for PAN/CNT composite fibers, slippage of polymers over each other and creation of defects and voids in structure resulted in higher overall porosity compared to freely spun fibers even at a jet stretch ratio of two. CNTs may act like impurities among polymer chains, reducing the structural integrity at jet stretch ratios of two and higher. As a result, the tolerance of PAN/CNT fibers against stretch forces inside coagulation bath was less than PAN fibers. For jet stretched fibers, *T_g_* of PAN/CNT fibers was also higher than neat PAN fibers (Table 2). This was due to lower mobility of PAN chains in the presence of CNTs, which may suggest the formation of an interphase between nanotubes and polymers.

### 3.3. Morphology and Microscopic Structure

#### 3.3.1. Shear Rate Effect

**Figure 3:**
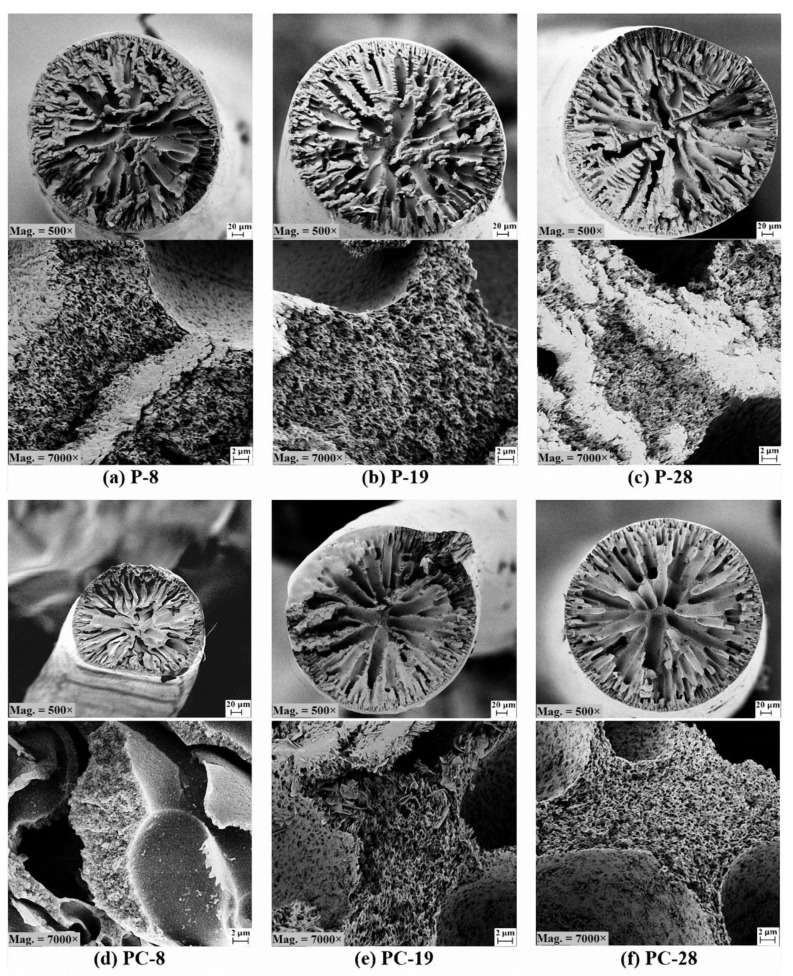
FESEM images from cross section and internal morphology of PAN and PAN/CNT fibers spun with different shear rates at magnifications of 500× and 7000×.

The FESEM images from cross section and internal morphology of PAN and PAN/CNT fibers spun at different shear rates are shown in Figure 3. No big morphological differences between PAN and PAN/CNT fibers could be observed. In general, the structure of fibers consisted of loose fibrils and finger like hollow pores in the radial direction of fibers. Formation of this structure is due to counter diffusion of solvent/nonsolvent and phase separation during coagulation [16]. The channels formed during counter diffusion of solvent/nonsolvent and the areas occupied by the liquid phase are responsible for formation of voids in fibrillar structure [16]. As the shear rate increased, the fibrillar parts and internal surfaces of finger-like voids became looser as a result of bigger jet swell (Figure 2). The outer surface of fibers—as it can be seen in Figure 3—were fairly smooth without grooves due to negligible stretching force inside the coagulation bath.

#### 3.3.2. Jet Stretch Effect

**Figure 4:**
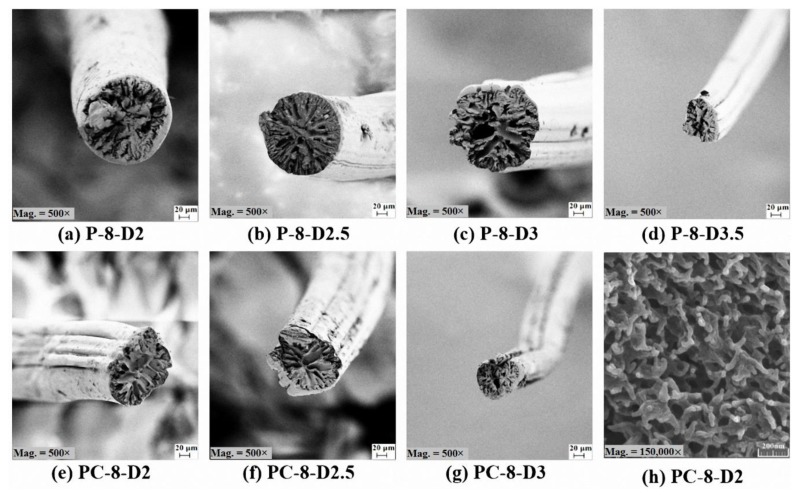
(**a**–**g**) FESEM images of PAN and PAN/CNT fibers spun at different jet stretch ratios at magnification of 500× (**h**) Internal morphology of PC-8-D2 fibers at magnification of 150,000×.

The effect of jet stretch on cross section shape and morphology of PAN and PAN/CNT fibers is shown in Figure 4. A significant decrease in fiber diameter by applying jet stretch was observed. Jet stretch ratio of two and higher changed the cross-section shape of PAN/CNT fibers from circular to an irregular shrunken form with numerous deep surface grooves. It confirmed that even a jet stretch ratio of two was higher than the optimum value for production of PAN/CNT composite fibers. However, the cross-section shape of PAN fibers was still circular at jet stretch ratios of 2 and 2.5. Deviation of cross-section shape from circular has an influence on the physical and mechanical properties of fibers [15,36]. The stress distribution is uniform in fibers with circular cross section, leading to improvement in mechanical strength [15,36], whereas fibers with non-circular cross section cannot tolerate high stretches in the following production stages due to stress concentration.

The outer surface of PAN reference fibers were smooth and without grooves at a jet stretch ratio of two, whereas fine grooves appeared on the surface at jet stretch ratio of 2.5. Surface grooves were caused by elongational forces applied on fibers inside the coagulation bath. The number and depth of these grooves grew with jet stretch ratio. For PAN/CNT composite fibers, however, the deep surface grooves could be observed even at jet stretch ratio of two, again confirming that PAN/CNT fibers were less tolerant for jet stretch compared to PAN fibers. Probably, the presence of CNTs prevented proper integration of structure under stretch. A jet stretch ratio of two and higher led to disintegration of structure and internal damages in PAN/CNT fibers. In addition, the reduction of internal friction in the presence of CNTs could lead to slippage of polymer chains over each other at high stretch ratios, creating damages in fibers structure. These damages grew with jet stretch ratio, where at jet stretch ratio of 3.5 filament self-breakage did not allow for fiber production.

In comparison with freely spun fibers, the pores in cross-section of jet stretched PAN and PAN/CNT fibers were smaller and narrower. The residence time of filaments in the coagulation bath was shortened by jet stretch, reducing the available time for nucleation and growth of voids, resulting in fibers with lower porosity [37,38]. Arbab et al. [37,39] has also observed more compact structure with smaller voids by increasing the jet stretch ratio in wet spinning of PAN fibers. In addition, the applied tension on fibers during jet stretch makes the fiber thread thinner, limits the mobility of polymer parts, and hinders the counter-diffusion of solvent/nonsolvent. Thus formation of big voids is avoidable to some extent. On the other hand, the already formed bigger voids can be stretched and shrunk when the thread thinning is taking place during stretching [16]. The calculated overall porosity of PAN fibers up to a jet stretch ratio of three in Table 2, also confirmed this argument. On the other hand, the overall porosity of PAN/CNT composite fibers increased by applying a jet stretch ratio of two and higher (Table 2). This could be a result of the presence of CNTs among polymer chains. In addition, the jet stretch ratios over the tolerance limit of PAN/CNT fibers caused structural defects and voids in the fibers. This observation confirmed the necessity of applying lower jet stretch ratios on PAN/CNT fibers compared to PAN fibers. When jet stretch ratio was very high (three for PAN/CNT fibers, 3 and 3.5 for PAN fibers), bigger voids were again obvious in the cross-section of fibers, confirming the damages to structure.

It was not possible to identify CNTs even at magnifications as high as 150,000× (Figure 4h). This could result from similar appearance of CNT individuals and their bundles with fibrillar form of porous as-spun PAN. Considering the smoother surface of CNTs compared to PAN fibrils, it could be deduced that CNTs may have been wrapped by polymer chains and an interphase might have been formed between PAN and CNT.

### 3.4. Crystalline Structure of Fibers

The supramolecular structure of coagulated as-spun fibers—i.e., crystallinity, crystallite size and crystal orientation—is directly related to mechanical properties of final fibers [28]. Therefore, it is important to study crystalline structure to understand the mechanical properties of fibers. 

#### 3.4.1. Shear Rate Effect

**Figure 5:**
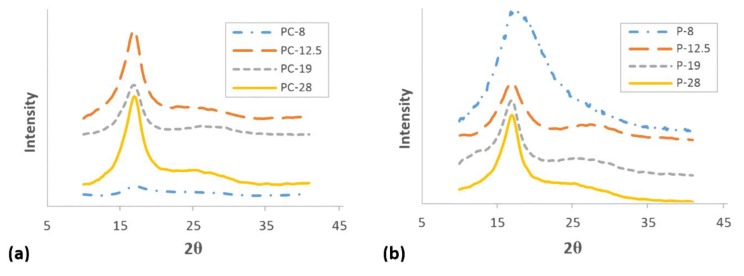
XRD patterns of fibers spun at different shear rates. (**a**) PAN/CNT fibers, (**b**) PAN fibers.

The effect of shear rate on X-ray diffraction patterns of PAN and PAN/CNT fibers are depicted in Figure 5. The data extracted from the XRD patterns after peak resolving are listed in Table 3. The intense peak at 2θ~17° was attributed to the diffraction from crystalline portion and the wide scattered diffraction at 2θ between 25°–30° was attributed to amorphous portions of fibers [28]. From XRD patterns it could be deduced that crystalline structure of PAN and PAN/CNT fibers was the same. Mai et al. [23] also observed the same crystal structure for PA66 fibers and their composites with MWCNT.

The intensity of diffraction peak at 2θ~17° for PAN/CNT fibers spun at shear rate of 8000 s^−1^ is very weak, indicating very low crystallinity degree (Figure 5a). The data extracted from XRD patterns of PAN/CNT fibers in Table 3 also shows large FWHM, small crystallite sizes (*L_c_*) and low crystallinity (*C*%). This peak for PAN fibers spun at shear rate of 8000 s^−1^ (P-8) was very wide, which made it very difficult to resolve to crystalline and amorphous portions (Figure 5b). This wide peak apparently indicates that amorphous phase was dominant in fibers. Therefore, the data obtained from this curve was not fully reliable and could only be used for comparison. However, it was clear that the crystallinity was low and the crystallite size was very small.

**Figure 6:**
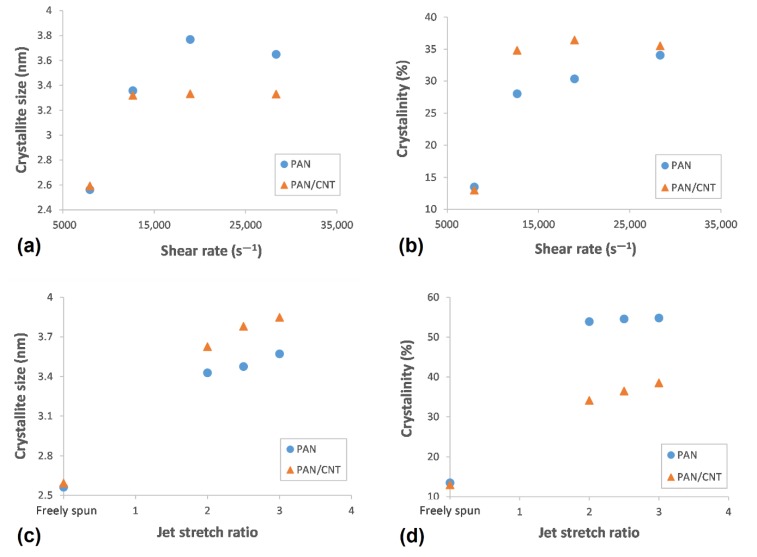
(**a**) The crystallite size and (**b**) crystallinity of PAN and PAN/CNT fibers as a function of shear rate, (**c**) The crystallite size and (**d**) crystallinity of PAN and PAN/CNT fibers as a function of and jet stretch ratio.

**Figure 7:**
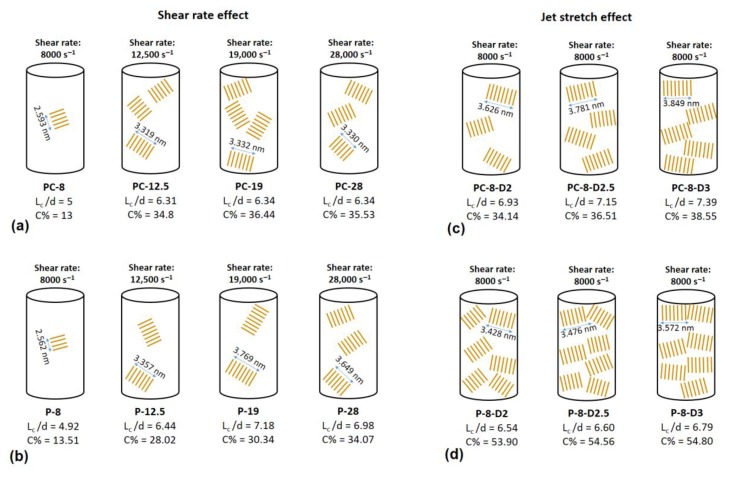
A schematic showing the changes in crystalline parameters of PAN and PAN/CNT fibers at different spinning conditions (**a**) PAN/CNT fibers at different shear rates, (**b**) PAN fibers at different shear rates, (**c**) PAN/CNT fibers at different jet stretch ratios, (**d**) PAN fibers at different jet stretch ratios.

Increasing the shear force on polymers can nucleate new crystals [23,40]. In addition, it can increase the alignment of polymers along the spinning direction [4,41], so they would have more possibility to be attached to crystal nuclei. Both reasons can develop the crystalline phase, resulting in crystallite size and crystallinity improvements. According to Table 3, the peak width for both PAN and PAN/CNT fibers was decreased with an increase in the shear rate, indicating the growth of crystals and increase of crystallite size (Figure 6). *L_c_*/*d*, which is a measure of average layers in crystallites [27], also grew with the shear rate, in which *d* stands for d-spacing of (*hkl*) reflections. This trend is shown schematically in Figure 7. 

At shear rate of 28,000 s^−1^, the highest shear force was applied on polymer chains leading to nucleation of crystals and the increase in crystallinity of PAN fibers to 34% (Table 3 and Figure 6). However, smaller crystallite size (*L_c_*: 3.649 nm) compared to shear rate of 19,000 s^−1^ (*L_c_*: 3.769 nm) showed that higher than optimal value of shear force probably caused the slippage of polymer chains over each other along the flow direction of spinning dope. Consequently, the possibility of attachment of chains to crystalline phase was less and the crystallite size was smaller. The average number of crystalline layers of PAN fibers spun at shear rate of 28,000 s^−1^ (i.e., 6.98) was also less than that of shear rate of 19,000 s^−1^ (i.e., 7.18). At lower shear rates, the possibility of nucleation of crystals was less, while the nucleated crystals were bigger due to attachment of more chains to the crystalline phase. 

On the other hand, both crystallite size and crystallinity were decreased in PAN/CNT composite fibers spun at shear rate of 28,000 s^−1^ (*L_c_*: 3.330 nm, *C*%: 35.53%) (Table 3, Figure 6). The presence of CNTs changed the response of polymer chains to shear force during the spinning process. It was easily recognizable by naked eyes that the viscosity of PAN/CNT spinning dope was less than PAN spinning dope. Mikolajczyk et al. [4] also reported the reduction in internal friction of PAN solution with addition of CNTs. At high shear rate of 28,000 s^−1^, the slippage of polymer chains in spinning dope with lower viscosity hindered the crystal growth and crystallinity enhancement.

Comparison of crystalline parameters of PAN and PAN/CNT fibers at different shear rates in Table 3 and Figure 6 revealed that PAN/CNT fibers had higher crystallinity but smaller crystallites with less crystalline layers. The higher crystallinity of PAN/CNT fibers also led to lower mobility of polymer chains compared to neat PAN fibers, resulting in higher *T_g_* values (Table 2). The increase in crystallinity can have a big effect on mechanical properties of PAN/CNT fibers, which is beyond the direct reinforcement resulted from load transfer [14]. The crystallite sizes of PAN and PAN/CNT fibers were almost identical up to the shear rate of 12,500 s^−1^. At higher shear rates, the crystallite sizes of PAN fibers exceeded those of PAN/CNT fibers. In the presence of CNTs, the nucleation of crystals under shear force was increased. Possibly the CNTs acted as nuclei for formation of new crystals with smaller size [23,40,42]. Polymer chains can interact with CNTs and nucleate on their surface [23,40]. Chae et al. [43] observed that CNTs are surrounded by a graphitic layer after carbonization of PAN/CNT fibers at low temperatures of 1100 °C. Formation of this graphitic layer can be indicative of ability of CNTs to act as templates for orientation and crystallization of polymer chains [43]. In the absence of CNTs in PAN reference fibers, however, fewer number of new crystals were formed, whereas the formed crystals grew more under shear force compared to PAN/CNT fibers. In the other words, shear force was more beneficial for nucleation of new crystals in PAN/CNT composite fibers and for growth of formed crystals in PAN reference fibers. 

**Figure 8:**
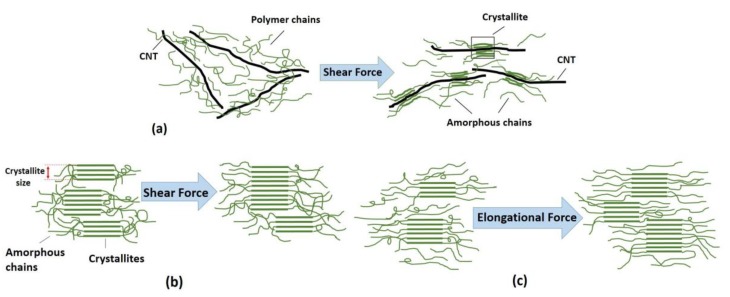
Schematics showing (**a**) possible mechanism for the effect of shear force on the interface between polymer and CNT, (**b**) the effect of shear force on crystallite size of PAN and PAN/CNT fibers, (**c**) the effect of elongational force on crystallite size and crystallinity of PAN fibers.

The extended-polymer chain conformation is the most desired structure for formation of fibers with high modulus and strength [40]. To induce extended chain crystallization, extensional force that is usually applied to polymer solution through shear flow is required [40]. Zhang et al. [40] observed that in PAN/single-walled CNT fibers with high concentration of PAN that has been under shear force, an interface in form of fibril-like crystals with extended chain is formed. Considering the production methods used in this research, i.e., applying shear force on PAN/CNT solution during preparation of spinning dope as well as during the passing of the spinning dope through the spinneret, could nucleate new crystals. Consequently, higher crystallinity in PAN/CNT fibers could be obtained compared to PAN fibers. The possible mechanism for the effect of shear force on crystal nucleation and crystallite size growth is shown in Figure 8a,b.

#### 3.4.2. Jet Stretch Effect

**Figure 9:**
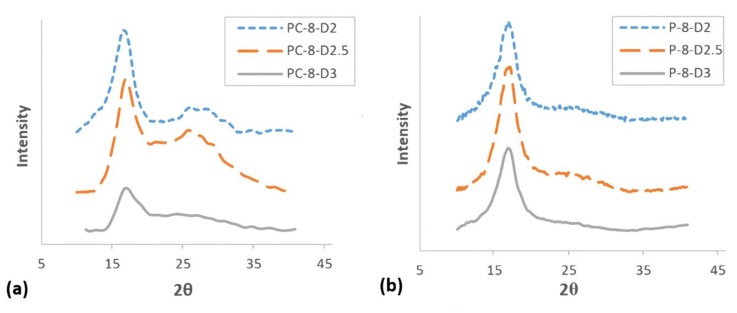
XRD patterns of fibers spun at different jet stretch ratios. (**a**) PAN/CNT, (**b**) PAN.

In Figure 9, the XRD patterns of PAN and PAN/CNT fibers spun under different jet stretch ratios are shown. The data extracted from these patterns after peak resolving are listed in Table 3. Applying jet stretch on fibers and increasing its ratio up to three decreased the width of crystalline peak at 2θ~17° for both PAN and PAN/CNT fibers. The crystallite size and crystallinity of jet stretched fibers were considerably higher than freely spun fibers at the same shear rate of 8000 s^−1^. The elongational force from application of jet stretch inside the coagulation bath increased the orientation of polymer chains along the fiber direction. Therefore, more polymer chains were placed in proper position to join the crystalline phase. Consequently, crystallite size and crystallinity increased (Table 3, Figure 6). 

When comparing crystal parameters of PAN and PAN/CNT fibers that were jet stretched inside the coagulation bath, it became obvious that the presence of CNTs was beneficial for the growth of crystallite size, whereas it had less effect on crystallinity improvement compared to PAN reference fibers. Namely, when jet stretch was applied on fibers in the coagulation bath, PAN/CNT fibers had fewer but bigger crystals compared to PAN reference fibers, in which the crystals were greater in number but in smaller sizes. Chai et al. [1] also observed bigger crystallite size and lower crystallinity of PAN fibers in presence of MWCNT. It showed the ability of PAN chains to nucleate new crystals under elongational force, compared to PAN/CNT fibers. CNTs apparently act as deterrent for nucleation of new crystals under high elongational force. The high jet stretch ratios applied in this research, may lead to overstretching of polymer chains in composite fibers and their slippage over each other, thus hindering the nucleation and formation of new crystals. However, polymer chains in PAN/CNT fibers could join the already formed crystals under elongational force, due to orientation of polymers along fiber direction. Overall, it is clear that CNTs had a different impact on crystalline structure of fibers under shear and elongational forces. Under shear force, CNTs induced nucleation of new crystals, whereas under elongational force nucleation of new crystals were hindered but the already formed crystals had grown bigger. To our knowledge, this key effect has not been studied and reported elsewhere.

Comparison of crystalline parameters of jet stretched and freely spun reference PAN fibers revealed that crystallinity of jet stretched fibers spun at shear rate of 8000 s^−1^ was considerably higher than fibers freely spun at highest shear rate of 28,000 s^−1^ (Table 3). However, the crystallite sizes of jet stretched fibers were not bigger than freely spun fibers at high shear rates of 19,000 and 28,000 s^−1^. These results showed the stronger effect of shear force on growth of crystallites and the bigger effect of elongational force on the crystallinity of PAN reference fibers. Probably under shear force, the crystallites in PAN fibers had the ability to join each other, whereas under elongational force polymer chains in amorphous phase were stretched and oriented along fiber direction, being more able to join the crystalline phase. This mechanism is schematically represented in Figure 8b,c. 

Unlike PAN fibers, the differences between crystallinity of PAN/CNT fibers freely spun at different shear rates or under jet stretch were not considerable (Table 3). Again, unlike PAN fibers, applying jet stretch on PAN/CNT fibers increased crystallite size compared to PAN/CNT fibers freely spun at different shear rates. This was because of the stronger effect of shear force on crystallinity of PAN/CNT composite fibers compared to elongational force. It may have resulted from the effect of CNTs and their bundles on hindering the extension and orientation of polymer chains under jet stretch inside the coagulation bath. It may have also been due to the minor effect of high jet stretch ratios used in this research on crystallinity of PAN/CNT fibers. As it was reported in Section 3.3.2 (Figure 4), jet stretch ratios used in this research were higher than tolerance limit of PAN/CNT fibers, causing damages to fiber structure and deviation of cross-section shape from regular circular form. The high porosity of jet stretched PAN/CNT fibers (Table 2) can also be a confirmation on slippage of chains over each other at high jet stretch ratios or prevention of long CNTs from free mobility and alignment of polymer chains, creating voids in the structure.

### 3.5. Mechanical Properties

**Figure 10:**
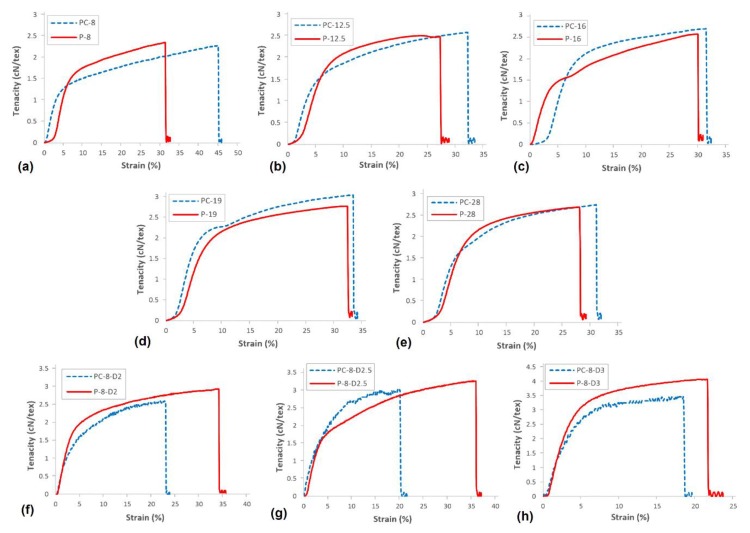
The tenacity–strain curves of PAN and PAN/CNT fibers spun at different shear rates and jet stretch ratios. (**a**) At shear rate of 8000 s^−1^, (**b**) At shear rate of 12,500 s^−1^, (**c**) At shear rate of 16,000 s^−1^, (**d**) At shear rate of 19,000 s^−1^, (**e**) At shear rate of 28,000 s^−1^, (**f**) At jet stretch ratio of 2, (**g**) At jet stretch ratio of 2.5, (**h**) At jet stretch ratio of 3.

Mechanical properties of PAN and PAN/CNT as-spun fibers at different conditions of shear and jet stretch are reported in Table 4. The tenacity–strain curves for PAN and PAN/CNT fibers spun at different shear rates and jet stretch ratios are presented in Figure 10. It needs to be mentioned that to study the effect of spinning parameters on the structure and properties of first fiber-form product obtained in the coagulation bath, the properties reported here were related to as-spun fibers. The post-drawing stage—as an important stage in improvement of fiber properties—was not performed on the reported fibers and is the focus of our ongoing research. The results of post drawing stage on the structure and properties of PAN/CNT fibers are to be presented in other research papers. 

#### 3.5.1. Shear Rate Effect

If jet stretch was not applied on fibers, the orientation of polymer chains that was improved inside the spinneret was almost lost after exiting from the spinneret. As a result, the improvement in mechanical strength with increase in shear rate was not considerable (Table 4). Considering the results obtained from XRD patterns in Section 3.4.1, the crystals nucleated under shear force remained in the fibers after exiting the spinneret. These crystals may be responsible for minor improvement in mechanical properties of fibers with shear rate. The highest mechanical strength for both PAN and PAN/CNT fibers were obtained at shear rate of 19,000 s^−1^ and the strength decreases at shear rate of 28,000 s^−1^. The fiber strength not only is influenced by orientation of macromolecules inside fibers, but also depends on intact molecular structure with fewer voids and defects and higher crystallinity [44,45]. It seems that the diminution of mechanical strength of PAN and PAN/CNT fibers at shear rate of 28,000 s^−1^ was a result of application of shear rates higher than optimum values. One of its consequences was higher jet swell (Figure 2) and eventually loss of orientation and mechanical properties of fibers. In addition, at higher shear rates the revolution of metering pump was faster, increasing the possibility of formation of bubbles inside the spinning dope while passing through the pump and spinning path. These bubbles can remain in the fiber as defects and act as a source of fiber failure under stretching force [31]. Furthermore, the slippage of polymer chains over each other at very high shear forces, which leads to smaller crystallite sizes and lower crystallinity, might be another reason for lower mechanical strength of fibers.

The presence of CNTs affects the mechanical properties of PAN fibers by changing the structure parameters such as orientation, crystallinity and crystallite size. Fei et al. [9] observed the improvement in mechanical compressive strength of polystyrene composite foams by addition of MWCNTs, which was gradually improved by increasing the MWCNT wt% up to 1%. Comparison of mechanical strength of PAN and PAN/CNT fibers spun at different shear rates showed that even having the higher porosity (Table 2), the strength of freely spun PAN/CNT fiber improved up to 20% compared of corresponding PAN fibers. This was a result of the reinforcing role of CNTs in the structure, suggesting the formation of an interphase between polymer and CNTs. It also showed the possibility of nucleation of new crystals templated by CNTs and enhancement of fiber crystallinity (Table 3). Moreover, due to reduction of elastic recovery of PAN chains in the presence of CNTs, PAN/CNT fibers can retain their orientation better, resulting in a decrease in the number of entanglements in the structure of as-spun fibers [30]. 

Similar to mechanical strength, Young’s modulus of PAN and PAN/CNT fibers did not have regular incremental or decreasing trend with shear rate (Table 4). Young’s modulus is influenced by crystallinity of structure, perfection of crystallites, alignment of crystals along fiber direction and orientation of polymer chains [46]. The most important factor in orientation of polymer chains and crystals in fibers is application of elongational force. In the absence of elongational force in freely spun fibers, the formed crystals under shear did not have proper orientation along fiber direction. Thus, the change in Young’s modulus of PAN and PAN/CNT fibers with shear rate was not significant.

The comparison between PAN and PAN/CNT fibers spun at different shear rates in Table 4 showed that the Young’s modulus of PAN/CNT as-spun fibers at all shear rates was up to 25% higher than that of PAN fibers. Due to the presence of CNTs, the crystalline structure of composite fibers has been improved through CNT templated crystallization. Possibly an interphase has been formed between CNTs and polymer chains leading to an improvement in stress transfer from soft polymer matrix to stiff CNTs. Consequently, Young’s modulus of as-spun PAN/CNT composite fibers was improved compared to PAN fibers even in the absence of elongational force in the coagulation bath, when orientation of polymers and crystals was not noticeable. 

The presence of CNTs in PAN fibers increased the strain at break of freely spun PAN/CNT composite fibers compared to PAN fibers (Table 4). The improvement in mechanical properties of composite fibers resulted from interaction between CNTs and PAN matrix. The conventional reinforcements for composites usually improve the modulus and strength at the expense of strain at break [1]. CNTs as reinforcements for PAN fibers, however, could improve strength, modulus and strain at break simultaneously, thus resulting in higher toughness up to 18% (Table 4), as Chae et al. [1] also reported in their research. 

#### 3.5.2. Jet Stretch Effect

**Figure 11:**
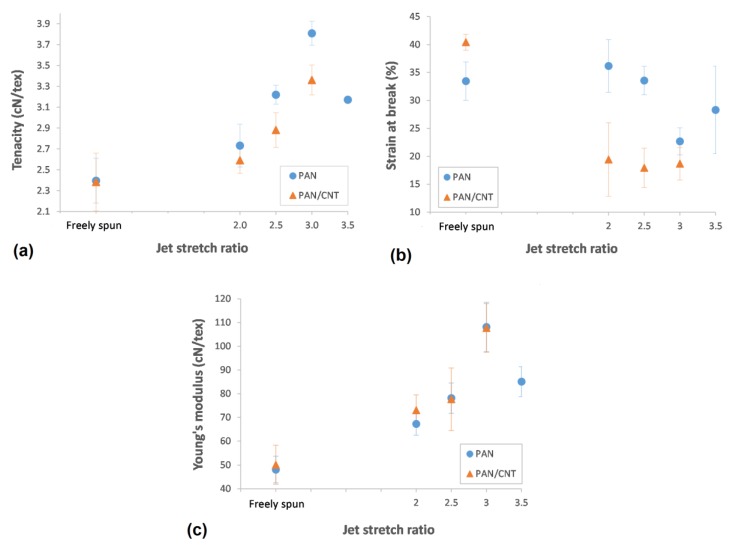
(**a**) Tenacity, (**b**) strain at break and (**c**) Young’s modulus of PAN and PAN/CNT fibers as a function of jet stretch ratio.

According to Table 4 and Figure 11, applying jet stretch to PAN and PAN/CNT fibers spun at shear rate of 8000 s^−1^ improved the strength of fibers compared to corresponding freely spun fibers. The elongational force applied on filaments inside the coagulation bath can open the random coils in polymer chains. It can reduce or eliminate the jet swell, enhancing the chain alignment and orientation, increasing the compactness of structure and eventually improving the crystallinity and mechanical properties of fibers. Internal micro-voids can become smaller as well [25,41]. The biggest improvement in mechanical strength, which was about 40% for PAN/CNT fibers and 60% for PAN fibers compared to corresponding freely spun fibers, was achieved at a jet stretch ratio of three. Investigation of crystalline structure of PAN fibers also showed the highest crystallinity and crystallite size at a jet stretch ratio of three. At a jet stretch ratio of 3.5, the strength of PAN fibers was reduced, showing that this jet stretch ratio was higher than tolerance limit of polymer chains, causing chain rupture and damages to structure. It could be seen from appearance of PAN fibers spun at jet stretch ratio of 3.5 that fibers split at many spots along the length of fibers. FESEM images also showed deep grooves on the surface of these fibers resulting from high stretching forces (Figure 4). The sudden porosity increase at this jet stretch ratio (Table 2) was another reason for the reduction in mechanical properties of PAN fibers at a jet stretch ratio of 3.5. 

Although the jet stretch ratios of two and higher improved the mechanical properties of PAN/CNT fibers compared to freely spun fibers at the same shear rate, it could be seen that higher shear rates had bigger influence on tensile strength (Table 4). These results confirm again that the optimal jet stretch ratios for PAN and PAN/CNT fibers were different. To the best of our knowledge, these results have not been reported elsewhere, since most research has focused on final PAN/CNT fibers than the early structure of as-spun PAN/CNT fibers. This understanding can be useful for designing the spinning process of composite fibers. The stretch ratios applied in this research were not optimized for improving the structure of PAN/CNT as-spun fibers as it was expected, due to the different focus of this study. 

Comparison of PAN and PAN/CNT fibers at different jet stretch ratios showed that the strength of jet stretched PAN/CNT as-spun fibers was about 5%–12% less than corresponding PAN fibers (Table 4, Figure 11). Applying jet stretch improved orientation and mechanical strength of PAN fibers more than PAN/CNT fibers due to following reasons: 1. Jet stretch ratios applied on PAN/CNT fibers were higher than optimum value. Due to lower viscosity of PAN/CNT spinning dope, the jet stretch ratio must be less than PAN fibers to increase the orientation of polymer chains without slippage of structural units over each other. 2. The cross-section shape of jet stretched PAN/CNT fibers changed from circular to an irregular form (Figure 4), which is in general not beneficial for mechanical properties of fibers due to stress concentration. 3. The presence of CNTs and their possible bundles and aggregations as well as the formed interface between CNTs and polymer chains, will probably reduce the free movement of polymers under stretching force, hindering the crystallinity enhancement and increasing the fiber porosity. Although increasing the jet stretch ratio seems economically beneficial and increases the production speed, one should take into account the differences in tolerance limit of PAN and PAN/CNT fibers for jet stretch, when designing the production line. Overall, it seems that lower jet stretch ratios were required for production of PAN/CNT fibers to retain and increase orientation, improve crystalline structure and crystal orientation, decrease the porosity and increase the mechanical strength without damaging the fiber structure. 

Application and increasing the jet stretch ratio increased the Young’s modulus of PAN and PAN/CNT as-spun fibers significantly. The highest Young’s modulus was obtained at a jet stretch ratio of three for both PAN and PAN/CNT fibers. As it was already mentioned in Section 3.4.2, the crystallinity was also the highest at jet stretch ratio of three (Table 3). Although the application of jet stretch did not improve the crystallinity of PAN/CNT fibers considerably compared to freely spun composite fibers at high shear rates, the Young’s modulus was still significantly higher than freely spun fibers. This revealed the importance of chain orientation for improvement of Young’s modulus. When chain orientation is increased, more primary chemical bonds are distributed along the fiber direction to tolerate the applied force, leading to an improvement in mechanical properties of fibers. For PAN fibers, Young’s modulus decreased significantly at a jet stretch ratio of 3.5. It confirmed that this stretch ratio was higher than the bearing limit of polymer chains, causing damages to structure, as confirmed in FESEM images in Figure 4.

Comparison of Young’s modulus of PAN and PAN/CNT fibers at different jet stretch ratios showed that despite significantly lower crystallinity (Table 3), lower tensile strength (Table 4) and higher porosity (Table 2) of jet stretched PAN/CNT fibers compared to PAN fibers, Young’s modulus of PAN/CNT as-spun fibers at jet stretch ratio of two was about 10% higher than PAN fibers. At higher jet stretch ratios, Young’s modulus of PAN/CNT fibers was almost the same as PAN fibers. For jet stretched fibers, multi walled CNTs have bigger effect on Young’s modulus than on tensile strength. Some researchers have shown that the development of crystalline interface between polymer and CNTs can improve the stress transfer between matrix and these CNTs [12,47]. It seems that applying jet stretch strengthened the interface between polymer and CNTs by increasing the orientation of polymers and CNTs along the fiber direction, leading to improvement in Young’s modulus of PAN/CNT fibers. However, applying jet stretch ratios higher than optimum values for composite fibers prevented maximum reinforcing capabilities of CNTs inside polymer structure from being obtained.

### 3.6. Electrical Properties

#### 3.6.1. Shear Rate Effect

**Figure 12:**
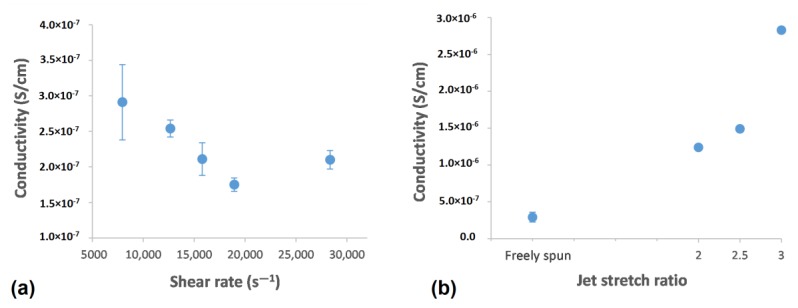
Electrical conductivity of PAN/CNT fibers as a function of (**a**) shear rate and (**b**) jet stretch ratio.

Electrical conductivity of PAN and PAN/CNT composite fibers as a function of shear rate and jet stretch ratio is illustrated in Figure 12. The electrical conductivity of neat PAN fiber was 1.80 × 10^−7^ S/cm. The electrical conductivity of composite fibers containing CNTs was increased up to about two times compared to PAN fibers. With an increase in shear rate, the electrical conductivity of PAN/CNT fibers decreased. When the shear rate increased, the fibers became thicker (Table 2). This thickening happened while the same percentage of CNTs existed inside fibers. As a result, the distance between CNTs became bigger and they had fewer connections with each other, which was deterrent for electrical conductivity of fibers. In addition, jet swell ratio was bigger at higher shear rates (Figure 2), resulting in bigger loss of orientation for polymers and CNTs. Therefore, CNTs might have been placed more randomly in the fibers instead of being aligned in fiber direction. This was unbeneficial for the transmittance of current along fibers and was considered another reason for lower conductivity of fibers at higher shear rates. Moreover, high porosity and the presence of macro voids in fibers could have prevented percolation between CNTs and reduced the current transmit through them.

#### 3.6.2. Jet Stretch Effect

Applying jet stretch on coagulating filament increased the electrical conductivity significantly (Figure 12b). This sudden increase was indicative of higher orientation of polymer chains and CNTs due to stretching force, which also improved the mechanical properties of fibers (Table 4). The highest electrical conductivity was obtained at a jet stretch ratio of three. The mechanical properties was also the highest at a jet stretch ratio of three. The results of mechanical and electrical properties confirmed that with an increase in polymer chain orientation at a jet stretch ratio of three, the CNTs were also oriented along fiber direction, increasing the current transmit through them, leading to higher electrical conductivity.

## 4. Conclusions

Wet spinning of PAN and PAN/CNT fibers was studied and the effect of different shear and stretching conditions on the structural, mechanical and electrical properties of as-spun fibers was investigated. The results showed different effects of CNTs on crystalline structure of fibers under shear and elongational forces. CNTs caused nucleation of new crystals under shear force, whereas under elongational force nucleation of crystals were hindered but the already formed crystals grew bigger. To our knowledge, this key effect has not been studied and reported elsewhere. In the absence of CNTs in PAN fibers, shear force influenced the crystal growth more, whereas the elongational force rather affected the crystallinity. At different shear rates, mechanical strength, Young’s modulus and strain at break of freely spun PAN/CNT fibers improved up to 20% compared to PAN fibers due to possible formation of interface between polymer and CNTs. At different jet stretch ratios, the presence of CNTs rather influenced the Young’s modulus than mechanical strength. Despite lower crystallinity and much higher porosity of composite fibers than PAN reference fibers at different jet stretch ratios, high Young’s modulus can still be obtained in PAN/CNT fibers. Microscopic observations and porosity estimation showed that the tolerance of PAN/CNT fibers for jet stretch inside a coagulation bath was less than PAN fibers. Understanding the effect of shear and elongational forces during spinning process on the structure and properties of PAN/CNT as-spun fibers can help to design the production line carefully, in order to obtain better physical and mechanical properties in final PAN/CNT precursor fibers. 

## Figures and Tables

**Table 1:** Denotation and production conditions of polyacrylonitrile (PAN) and PAN/carbon nanotube (CNT) fibers.

Spinning Condition	Sample Names		Shear Rate (s^−1^)	Jet Stretch Ratio
	PAN/CNT	PAN		
Freely spun	PC-8	P-8	8000	-
	PC-12.5	P-12.5	12,500	-
	PC-16	P-16	16,000	-
	PC-19	P-19	19,000	-
	PC-28	P-28	28,000	-
Jet stretched	PC-8-D2	P-8-D2	8000	2
	PC-8-D2.5	P-8-D2.5	8000	2.5
	PC-8-D3	P-8-D3	8000	3
	PC-8-D3.5	P-8-D3.5	8000	3.5

**Table 2:** Physical properties of PAN and PAN/CNT fibers at different spinning conditions.

Spinning Condition	Sample Name	Diameter (μm)	Linear Density (tex)	*T_g_* (°C)	Porosity (%)	Sample Name	Diameter (μm)	Linear Density (tex)	*T_g_* (°C)	Porosity (%)
Freely spun	PC-8	275.0 ± 11	15.1 ± 0.8	112.2	78.5	P-8	335.4 ± 13	28.2 ± 1.7	106.3	73.1
	PC-12.5	309.0 ±15	20.0 ± 0.2	-	77.5	P-12.5	368.7 ± 18	34.8 ± 0.7	-	72.5
	PC-16	329.8 ± 7	21.4 ± 0.4	-	78.9	P-16	375.2 ± 17	36.3 ± 1.1	-	72.3
	PC-19	343.6 ± 9	22.3 ± 0.6	110.5	79.7	P-19	378.2 ± 16	36.4 ± 0.3	95	72.6
	PC-28	352.1 ± 13	26.0 ± 0.5	-	77.5	P-28	397.9 ± 18	39.3 ± 0.1	-	73.3
Jet stretched	PC-8-D2	147.9 ± 10	4.0 ± 0.3	98.5	80.3	P-8-D2	185.5 ± 11	10.0 ± 0.5	94.1	68.7
	PC-8-D2.5	138.0 ± 15	2.8 ± 0.4	-	84.1	P-8-D2.5	164.2 ± 14	7.3 ± 0.4	-	70.7
	PC-8-D3	91.3 ± 11	1.4 ± 0.2	-	85.4	P-8-D3	153.3 ± 22	6.2 ± 0.8	-	71.5
	PC-8-D3.5	-	-	-	-	P-8-D3.5	89.5 ± 18	1.7 ± 0.2	-	76.9

*T_g_*: Glass transition temperature.

**Table 3:** Crystalline parameters of PAN and PAN/CNT fibers after peak resolving.

Spinning Condition	Sample Name	Angle (2θ)	FWHM (2θ)	*d* (A)	*L_c_* (nm)	*L_c_/d*	*C*%	Sample Name	Angle (2θ)	FWHM (2θ)	*d* (A)	*L_c_* (nm)	*L_c_/d*	*C*%
Freely spun	PC-8	17.09	3.07	5.185	2.593	5.00	13.01	P-8	17.02	3.10	5.206	2.562	4.92	13.51
	PC-12.5	16.85	2.39	5.256	3.319	6.31	34.83	P-12.5	17.01	2.3	5.209	3.357	6.44	28.02
	PC-19	16.85	2.38	5.257	3.332	6.34	36.44	P-19	16.88	2.11	5.248	3.769	7.18	30.34
	PC-28	16.90	2.39	5.252	3.330	6.34	35.53	P-28	16.94	2.18	5.229	3.649	6.98	34.07
Jet stretched	PC-8-D2	16.93	2.19	5.233	3.626	6.93	34.14	P-8-D2	16.91	2.32	5.240	3.428	6.54	53.90
	PC-8-D2.5	16.76	2.10	5.286	3.781	7.15	36.51	P-8-D2.5	16.82	2.29	5.266	3.476	6.60	54.56
	PC-8-D3	17.00	2.06	5.210	3.849	7.39	38.55	P-8-D3	16.85	2.22	5.257	3.572	6.79	54.80

*d*: d-spacing

**Table 4:** Mechanical properties of PAN and PAN/CNT fibers at different shear rates and jet stretch ratios.

Spinning Condition	Sample Name	Tenacity (cN/tex)	Strain at Break (%)	Young’s Modulus (cN/tex)	Toughness (cN/tex)	Sample Name	Tenacity (cN/tex)	Strain at Break (%)	Young’s Modulus (cN/tex)	Toughness (cN/tex)
Freely spun	PC-8	2.38	40.41	50.09	0.72	P-8	2.39	33.45	48.07	0.63
	PC-12.5	2.50	32.11	49.17	0.60	P-12.5	2.43	27.97	47.49	0.56
	PC-16	2.70	30.09	54.62	0.63	P-16	2.48	29.25	51.12	0.60
	PC-19	3.00	30.04	57.66	0.68	P-19	2.82	28.88	49.16	0.59
	PC-28	2.67	32.70	47.34	0.63	P-28	2.66	28.73	46.56	0.56
Jet stretched	PC-8-D2	2.59	19.43	73.04	0.38	P-8-D2	2.73	36.17	67.22	0.80
	PC-8-D2.5	2.88	17.95	77.63	0.41	P-8-D2.5	3.22	33.56	78.14	0.85
	PC-8-D3	3.36	18.68	107.71	0.52	P-8-D3	3.81	22.67	108.14	0.57
	PC-8-D3.5	-	-	-	-	P-8-D3.5	3.17	28.29	85.09	0.76
